# A marked enhancement of a BLOC‐1 gene, *pallidin*, associated with somnolent mouse models deficient in histamine transmission

**DOI:** 10.1111/cns.13995

**Published:** 2022-10-18

**Authors:** Laurent Seugnet, Christelle Anaclet, Magali Perier, Jean‐François Ghersi‐Egea, Jian‐Sheng Lin

**Affiliations:** ^1^ Integrative Physiology of the Brain Arousal Systems, Lyon Neuroscience Research Center, INSERM U1028, CNRS UMR 5292 Claude Bernard University Lyon 1 Bron France; ^2^ Department of Neurological Surgery University of California, Davis School of Medicine Sacramento USA; ^3^ Fluids and Barriers of the Central Nervous System, Lyon Neuroscience Research Center, INSERM U1028, CNRS UMR 5292 Claude Bernard University Lyon 1 Bron France

## CONFLICT OF INTEREST

Dr. Lin is an Editorial Board member of CNS Neuroscience and Therapeutics and a coauthor of this article. To exclude any bias, he was not involved in any editorial decision‐making related to the acceptance of this article for publication.

Histamine and orexin (or hypocretin) neurons act distinctly and synergistically in wake control. A double knockout mouse genotype lacking both histamine and orexins shows all sleep disorders of human narcolepsy. We identified in this mouse brain a sharp upregulation of a BLOC‐1 gene, pallidin, that is selectively associated with a deficient histamine neurotransmission and dramatic changes in the balance of cholinergic and aminergic systems in mice as well as an enhanced sleep in drosophila. This study demonstrates potential sleep disorders‐associated compensatory mechanisms with pallidin as a novel biomarker.

The maintenance of wakefulness requires a complex brain arousal network made up of various neurotransmitters and neuropeptides. Using knockout (KO) mouse models we have previously shown that histamine (HA) and orexin (Ox, also called hypocretin) neurons act distinctly and synergistically in terms of wake control.^1^, ^2^ An impaired histaminergic neurotransmission is associated with sleepiness in animal models and human sleep disorders while the lack of Ox neuropeptides constitutes a direct cause of narcolepsy, a neurological disease characterized by sleepiness and cataplexy.^1^, ^2^ We have generated a double KO mouse genotype lacking both HA and Ox [*Histidine decarboxylase (hdc, HA‐synthetizing enzyme)‐Orexin* KO, referred to as *HO*‐KO] which shows all phenotypes of human narcolepsy such as sleepiness, hypersomnia, sleep‐onset rapid eye movement and cataplexy‐like episodes, EEG hypersynchronization and marked obesity.^3^ This mouse strain constitutes therefore a complete murine model of narcolepsy. In order to identify the consequences on gene expression of sleep disorders and to uncover novel molecular and cellular processes involved in sleep–wake control, we performed transcriptomic profiling in the frontal cortex of *HO*‐KO mice and their wild‐type littermates.

**FIGURE 1:**
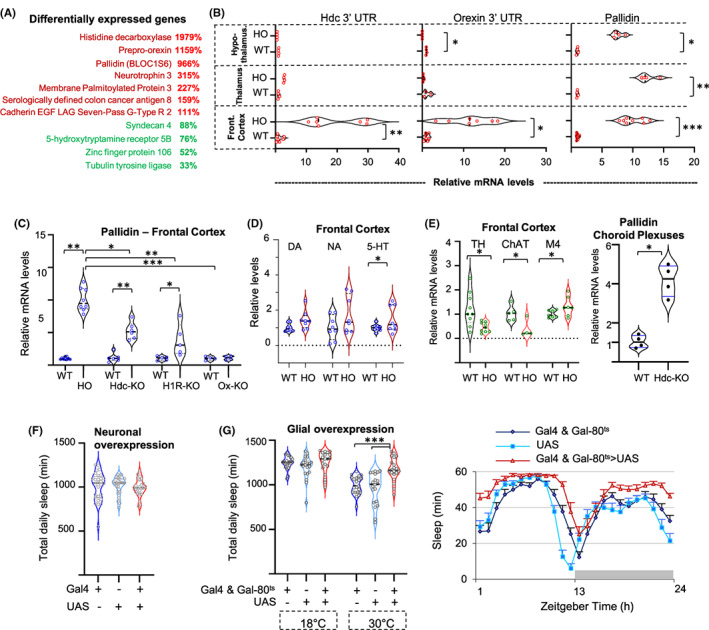
(A) Selection of differentially expressed genes in *HO*‐KO mice, confirmed by QPCR. Relative expression as % of controls is shown (*N* = 3–7, *p* < 0.05). (B) Relative expression levels as % of controls in the hypothalamus (top row), thalamus (middle row), and frontal cortex (bottom row) of *HO*‐KO mice, for the 3′ untranslated part of the truncated transcripts of *Histidine decarboxylase* (*Hdc*) and *Orexin* in the KO. Right: expression of *pallidin* in the same brain regions. *N* = 4–8. (C) Relative expression levels of *pallidin* transcripts in the frontal cortex HO‐KO compared to *Hdc*, *Histamine H1 Receptor* (*H1R*), and *Orexin* (*Ox*) single KO mice. *N* = 4–8. (D) Left: levels of Noradrenaline (NA), Dopamine (DA), and Serotonin (5‐HT) as measured by HPLC in the frontal cortex of *HO*‐KO mice, right: relative transcripts levels for the *tyrosine hydroxylase* (TH), Choline acetyltransferase (ChAT), and Muscarinic acetylcholine receptor M4 (M4) genes in the frontal cortex of *HO*‐KO mice (*N* = 4–9). (E) Relative expression levels of *pallidin* transcripts in the choroid plexuses of *Histidine decarboxylase* single KO mice. (F, G) Overexpression of *pallidin* in *Drosophila*. The experimental flies are in pink; the genetic controls are in blue. *N* = 20–21. (F) Overexpression of *pallidin* in neurons, the elav‐GeneSwitch construct was used as Gal4. (G) Overexpression of *pallidin* in all glial cells using the Gal4‐UAS system. The Repo‐Gal4 transgene was combined with the Tub‐Gal80^ts^ to inhibit Gal4 activity at 18°C. Flies were transferred at 30°C to induce the expression of *pallidin*. Left: total daily sleep, right: hourly sleep curve of the first 24 h spent at 30°C. *p < 0.05; **p < 0.005; ***p < 0.0005. See Appendix S1 for additional details and statistics.

We identified differentially expressed genes in this double mutant mouse that potentially reflect unidentified mechanisms controlling sleep and wakefulness (Appendix S1 and Table S1). Figure 1A shows a subset of these genes confirmed by independent quantitative PCR (QPCR). The non‐protein coding Hdc transcript was highly upregulated likely because of a negative feedback regulation between HA release and *Hdc* expression^4^ (Figure 1B). We obtained similar findings for the *Ox* non‐protein coding transcript (Figure 1B, middle). Interestingly this regulation was not uniform in the brain, indicating local compensatory regulatory mechanisms on neurotransmission (Figure 1B). The expression of genes not previously associated with sleep–wake regulation was also affected, notably *pallidin* (also called *BLOC1S6*), a gene coding a major subunit of the Biogenesis of Lysosome‐related Organelles Complex‐1 (BLOC‐1), that displayed a massively enhanced expression (>900%), in the frontal cortex as well as in the hypothalamus and thalamus (Figure 1B). To determine whether the upregulation of *pallidin* results from a HA or Ox deficiency or both, we performed QPCR in single KO mice lacking either *Hdc* or *Ox*. We found that enhanced *pallidin* expression occurred only in *Hdc*‐KO mice and not in *Ox*‐KO mice, indicating a HA‐dependent upregulation. We further found that *pallidin* expression was also highly upregulated in KO mice lacking postsynaptic HA H1‐receptor (Figure 1C), confirming that the lack of HA neurotransmission plays a key role in the regulation of this gene. Nevertheless, the consequences on gene expression following deletion of other HA receptors, notably the postsynaptic H2 receptor, remain to be investigated. The Ox transmission does not seem to affect *pallidin* expression. Yet, *pallidin* expression was markedly higher in the *HO*‐KO than in *Hdc*‐KO mice (Figure 1C), suggesting that the abnormal sleep phenotypes associated with Ox deficiency could contribute to the *pallidin* upregulation.

The BLOC‐1 complex is involved in protein trafficking among different endosomal compartments and has been linked to schizophrenia and cognitive performance.^5^ Indeed, BLOC‐1 genes play important roles in neuronal functions and in particular neurotransmission.^5^ In addition, it has been reported that *pallidin* could regulate transport at the blood–brain barrier and thus impact monoamine synthesis, and in particular that of serotonin.^6^ In line with this hypothesis, we found a marked increase in serotonin levels (Figure 1D), a change in expression of the *5HT‐5B* receptor gene (Figure 1A), and a significant decrease in mRNA levels for *Tyrosine Hydroxylase* in the frontal cortex of *HO*‐KO mice (Figure 1D). *Choline acetyltransferase* mRNA levels are also decreased, while muscarinic receptor M4 is higher in *HO*‐KO mice indicating that the cholinergic system is also upregulated (Figure 1D), consistent with findings seen with sleep regulation in H1‐receptor KO mice.^2^ HDC activity and HA transmission are present in non‐neuronal cell types of the brain, notably in the different blood–brain interfaces, including the choroid plexuses which form the blood‐CSF barrier, where HA affects gene expression.^7^, ^8^ We thus evaluated *pallidin* mRNA expression in the choroid plexuses of *Hdc*‐KO mice, and found that it was upregulated in these non‐neuronal cells (Figure 1E) as in the frontal cortex (Figure 1B).

A study based on locomotor activity suggested that *pallidin* could be involved in sleep–wake regulation in the mouse,^9^ yet, direct evidence is lacking. The role of *pallidin* in sleep–wake regulation remains, therefore, to be explored, in particular using conditional, cell type‐specific KO approaches. Yet, such attempt is currently highly challenging in mammals given the broad expression of *pallidin* in numerous cells types in the brain and periphery and currently the lack of flexible tools to selectively vary *pallidin* expression. We thus turned to the *Drosophila* model, which is intensively used in sleep–wake research. All the genes involved in BLOC‐1 function are conserved in *Drosophila*, with similar interactions and functions as those identified in mammals.^10^ We tested neuronal and non‐neuronal function for *pallidin* using pharmacological or heat‐inducible transgenes Gal4‐UAS systems to overexpress *pallidin* (Figure 1F,G). In *Drosophila*, glial cells regulate neurotransmission and control the exchanges between the brain and circulating fluid, the hemolymph, fulfilling a function similar to the blood–brain barrier in mammals. We found that overexpression of *pallidin* in neurons did not produce any detectable effect (Figure 1F) while that in the glia using a heat‐inducible system significantly enhanced sleep (Figure 1G). In contrast, in a separate report on *Drosophila*, we found a wake‐promoting effect following downregulation of *pallidin*.^11^

*Pallidin* appeared among the most dramatically upregulated genes in the brain of mice deficient for HA transmission and characterized by a somnolent phenotype. Moreover, we found that overexpression of *pallidin* in *Drosophila* glia results in increased sleep time. Therefore, *pallidin* upregulation in *HO*‐KO and *Hdc*‐KO is likely linked to the altered control of sleep–wake in these somnolent mice. Gene expression dosage appears to be a critical factor in the stability of the BLOC‐1 complex^5^ both in mammals and *Drosophila*. If such is the case in the mammalian blood–brain interfaces, the upregulation of *pallidin* may significantly modulate the functionality of BLOC‐1. The signaling pathway leading to this transcriptional response together with its potential impact on sleep–wake regulation remain to be identified. HA transmission has been shown to regulate the blood–brain interfaces^7^ and ependymal cells, the circumventricular organs, and the choroid plexuses are in close proximity with HA terminals. Interestingly, all of the latter cells display dramatically enhanced *c‐fos* expression in response to acute pain induced by formalin injection, and in some cases even to saline injection, in *Hdc*‐KO mice,^8^ suggesting that the lack of HA transmission enhances transcriptional responses. In *Drosophila*, HA neurons modulate sleep–wake via chloride‐gated channels, and no metabotropic HA receptor has so far been identified, indicating that the interactions between the HA system and *pallidin* differ from those in mammals. Nevertheless, we found that increasing *pallidin* expression in *Drosophila* glial cells increased sleep while that decreasing it enhanced waking,^11^ effects that would be potentially linked to the transport of monoamines precursors, since monoamines such as serotonin and dopamine are major evolutionary conserved sleep–wake regulators and since in particular serotonin levels and *TH* mRNA were markedly affected in HO‐KO mice.

The differentially expressed genes identified in this study suggest widespread compensatory mechanisms upon impaired HA transmission. The unexpectedly sharp upregulation of *pallidin* might constitute part of such adaptive mechanisms allowing the brain to compensate for the lack of HA. Alternatively, this upregulation could be directly linked to the sleepiness caused by the deficient HA transmission. These questions open future investigations. In any case, *pallidin*, easily quantifiable by PCR, might constitute a biomarker of sleepiness in animals and humans. Similarly, studies on whether and how the identified changes in gene expression are involved in the regulation of neurotransmitter systems, notably acetylcholine, monoamines, and/or metabolism, should open new avenues in sleep–wake research.

## Supporting information


Appendix S1

Table S1

## Data Availability

Data will be made availabe on request.

## References

[1] Anaclet C , Parmentier R , Ouk K , et al. Orexin/hypocretin and histamine: distinct roles in the control of wakefulness demonstrated using knock‐out mouse models. J Neurosci. 2009;29(46):14423‐14438. 19923277 10.1523/JNEUROSCI.2604-09.2009 PMC2802289

[2] Parmentier R , Zhao Y , Perier M , et al. Role of histamine H1‐receptor on behavioral states and wake maintenance during deficiency of a brain activating system: a study using a knockout mouse model. Neuropharmacology. 2016;106:20‐34. 26723880 10.1016/j.neuropharm.2015.12.014

[3] Anaclet C , Ouk K , Guidon G , et al. Complementary and synergistic control of wakefulness by orexins and histamine, demonstrated using a double knockout mouse model. Sleep. 2010;33(Suppl):A47.

[4] Gondard E , Anaclet C , Akaoka H , et al. Enhanced histaminergic neurotransmission and sleep‐wake alterations, a study in histamine H3‐receptor knock‐out mice. Neuropsychopharmacology. 2013;38(6):1015‐1031. 23303066 10.1038/npp.2012.266 PMC3629391

[5] Ghiani CA , Dell'Angelica EC . Dysbindin‐containing complexes and their proposed functions in brain: from zero to (too) many in a decade. ASN Neuro. 2011;3(2):AN20110010. 10.1042/AN20110010 PMC3155195 21504412

[6] Cotzias GC , Tang LC , Miller ST , Sladic‐Simic D , Hurley LS . A mutation influencing the transportation of manganese, L‐dopa, and L‐tryptophan. Science. 1972;176(4033):410‐412. 5026160 10.1126/science.176.4033.410

[7] Haas HL , Sergeeva OA , Selbach O . Histamine in the nervous system. Physiol Rev. 2008;88(3):1183‐1241. 18626069 10.1152/physrev.00043.2007

[8] Palkovits M , Deli MA , Gallatz K , Tóth ZE , Buzás E , Falus A . Highly activated c‐fos expression in specific brain regions (ependyma, circumventricular organs, choroid plexus) of histidine decarboxylase deficient mice in response to formalin‐induced acute pain. Neuropharmacology. 2007;53(1):101‐112. 17544458 10.1016/j.neuropharm.2007.04.001

[9] Lee FY , Wang HB , Hitchcock ON , et al. Sleep/wake disruption in a mouse model of BLOC‐1 deficiency. Front Neurosci. 2018;12:1‐21. 30498428 10.3389/fnins.2018.00759 PMC6249416

[10] Cheli VT , Daniels RW , Godoy R , et al. Genetic modifiers of abnormal organelle biogenesis in a *Drosophila* model of BLOC‐1 deficiency. Hum Mol Genet. 2010;19(5):861‐878. 20015953 10.1093/hmg/ddp555 PMC2816613

[11] Li H , Aboudhiaf S , Parrot S , et al. *Pallidin* function in drosophila surface glia regulates sleep and is dependent on amino acid availability. Published online August 1, 2022: 2022.05.03.490434. 10.1016/j.celrep.2023.113025 37682712

